# Anti-Osteoporosis Effects of the *Eleutherococcus senticosus*, *Achyranthes japonica*, and *Atractylodes japonica* Mixed Extract Fermented with Nuruk

**DOI:** 10.3390/nu13113904

**Published:** 2021-10-30

**Authors:** So Young Eun, Yoon-Hee Cheon, Gyeong Do Park, Chong Hyuk Chung, Chang Hoon Lee, Ju-Young Kim, Myeung Su Lee

**Affiliations:** 1 Musculoskeletal and Immune Disease Research Institute, School of Medicine, Wonkwang University, 460 Iksandae-ro, Iksan 54538, Korea; eunsoyg@hanmail.net (S.Y.E.); hanleuni@naver.com (Y.-H.C.); rudeh2508@naver.com (G.D.P.); taylorchung@hanmail.net (C.H.C.); lch110@wku.ac.kr (C.H.L.); 2 Division of Rheumatology, Department of Internal Medicine, Wonkwang University Hospital, 460 Iksandae-ro, Iksan 54538, Korea

**Keywords:** Vigeo, nuruk fermentation, osteoclasts, bone resorption, osteoporosis

## Abstract

Vigeo is a mixture of fermented extracts of *Eleutherococcus senticosus* Maxim (ESM), *Achyranthes japonica* (Miq.) Nakai (AJN), and *Atractylodes japonica* Koidzumi (AJK) manufactured using the traditional Korean nuruk fermentation method. Although the bioactive effects of ESM, AJN, and AJK have already been reported, the pharmacological effects of Vigeo have not been proven. Therefore, in this study, we investigated whether Vigeo had inhivitory effects on lipopolysaccharide (LPS)-induced inflammatory bone loss in vivo and receptor activator of nuclear factor-B ligand (RANKL)-induced osteoclastogenesis and the related mechanism in vitro. Vigeo administration conferred effective protection against bone loss induced by excessive inflammatory response and activity of osteoclasts in LPS-induced inflammatory osteoporosis mouse model. In addition, Vigeo significantly suppressed the formation of tartrate-resistant acid phosphatase-positive osteoclasts induced by RANKL and inhibited F-actin formation and bone resorbing activity without any cytotoxicity. Moreover, Vigeo significantly inhibited RANKL-induced phosphorylation of p38, ERK, JNK, IκB, and AKT and degradation of IkB. Additionally, Vigeo strongly inhibited the mRNA and protein expression of c-FOS and NFATc1 and subsequently attenuated the expression of osteoclast specific marker genes induced by RANKL. We demonstrated for the first time the anti-osteoporosis effect of Vigeo, suggesting that it could be a potential therapeutic candidate for the treatment of osteoclast-mediated inflammatory bone diseases.

## 1. Introduction

Osteoporosis is a common disease caused by problems with the bone remodeling process and is characterized by a reduction in bone mass and bone microstructural alterations [1]. Although the pathogenesis of bone remodeling is not completely understood, it is clear that an imbalance in bone remodeling, osteoporotic fractures, and vertebral and hip fractures are associated with morbidity and increased mortality [1].

In order to counteract the side effects of osteoporosis drugs currently in use, many researchers have tried to develop new treatments for bone metabolic disorders based on natural plant-derived compounds. The use of plants for treatment of several diseases has a long history [2,3]. Numerous plant extracts have been investigated for their anti-osteoporosis activity, and some have been reported to have potent activity [4,5]. In addition, fermentation with plant products may further enhance the functional properties of medicinal plants or attenuate their toxicity and side effects [6,7]. In the fermentation process, complex substances are broken down into smaller molecules by microorganisms to generate stable products, which improves their pharmacological efficacy for disease prevention [8].

Vigeo is a fermented combined extract of *Eleutherococcus senticosus* Maxim (ESM), *Achyranthes japonica* (Miq.) Nakai (AJN), and *Atractylodes japonica* Koidzumi (AJK), using the traditional Korean nuruk fermentation method. ESM, also called Siberian ginseng, has been reported to protect the bone from damage as well as to exert an anti-osteonecrotic effect in vitro and in vivo [9]. Notably, treatment with ESM may potentially overcome the effects of inflammation in the bone [10], as ESM can reduce calcium and hydroxyproline excretion in glucocorticoid-induced osteoporotic model [11]. Moreover, a recent study demonstrated that ESM extract has an inhibitory effect on RANKL-induced osteoclast formation [12]. AJN is traditional medicine in Korea, Japan, and China, used as a remedy for pain control and improvement of functional disorders in patients with osteoarthritis [13]. AJN is a herbal medicine traditionally used for the treatment of obesity and related complications [14], reported to have anti-inflammatory [15], anti-arthritic [16], and antioxidant properties [17] and. AJK also exhibits a wide variety of bioactive properties, including anti-inflammatory [18], antioxidant [19], and anti-obesity activities [20]. However, the pharmacological effects of the combination of the ESM, AJN, and AJK extracts have not been demonstrated previously. Thus, in the present study, the beneficial effects of the nuruk fermented extract mix, Vigeo, on bone metabolism were studied for the first time.

In this study, we investigated the effect of Vigeo on the lipopolysaccharide (LPS)-triggered bone loss model by measuring bone loss, osteoclast activation, and bone resorption content. To support the promising in vivo effect of Vigeo, we also examined the inhibitory effects of Vigeo on receptor activator of nuclear factor-B ligand (RANKL)-stimulated osteoclast activation and the underlying mechanisms *in vitro*.

## 2. Materials and Methods

### 2.1. Preparation of Vigeo

Vigeo^®^ was supplied by PANAX BIO (Panax Bio Co., Ltd., Nonsan, Korea). Briefly, Vigeo was manufactured as follows: well washed and dried *Eleutherococcus senticosus* Maxim (135 g), *Achyranthes japonica* (Miq.) Nakai (78 g), and *Atractylodes japonica* Koidzumi (78 g) were extracted with hot water for 180 min using a Kyungseo extractor (COSMOS-660, Kyungseo E&P Co., Ltd., Incheon, Korea). Fresh yeast, rich nuruk, and popped rice were prepared for nuruk fermentation. After mixing rice nuruk (1 kg) with fresh yeast (4 g) and distilled water (1.5 L), the mixture was fermented for 96 h. Then, popped rice (3 kg) and a combined extract (hot water extract) were mixed and fermented at 26 °C for 15 days. The nuruk fermented extract mixture (Vigeo) was freeze-dried and then dissolved in distilled water according to the required concentration.

### 2.2. Reagents and Antibodies

Recombinant human RANKL and human macrophage colony-stimulating factor (M-CSF) were purchased from PeproTech EC, Ltd. (London, UK). The β-actin antibody was obtained from Sigma-Aldrich (St. Louis, MO, USA). Specific antibodies against phospho-p38, p38, phospho-ERK 1/2, ERK 1/2, phospho-JNK, JNK, phospho-IκB, phospho-AKT, and AKT, c-FOS, nuclear factor of activated T cells c1 (NFATC1), and IκB were purchased from Santa Cruz Biotechnology (Santa Cruz, CA, USA). Alpha minimal essential medium (α-MEM), fetal bovine serum (FBS), and penicillin-streptomycin solution were purchased from Gibco BRL (Grand Island, NY, USA).

### 2.3. Mice Care and Ethics Statement

The mice were tested by purchasing five-week-old males of the imprinting control region (ICR) mice (weighing 35 ± 2 g) from Samtako Co., Ltd. (Osan, Korea). All mice were fed a normal diet and provided free access to water, maintained at an ambient temperature of 22–24 °C and relative humidity of 55–60% under a 12 h:12 h light/dark cycle in a pathogen-free environment. Mouse studies were approved by the Institutional Animal Care and Use Committee (IACUC) of Wonkwang University (Permit number: WKU-21-02), Republic of Korea, and conducted as per the standard guidelines. The mice were monitored daily to check their health status.

### 2.4. LPS-Mediated Bone Loss Mouse Model

Five-week-old male ICR mice were randomly divided into four groups: control (*n* = 6), LPS (5 mg/kg) treated (*n* = 6), LPS (5 mg/kg) + Vigeo (100 mg/kg) treated (*n* = 6), and LPS (5 mg/kg) + Vigeo (200 mg/kg) treated (*n* = 6). Vigeo or PBS was administered orally every 9 days before the first LPS injection. Mice were intraperitoneally injected with LPS (10 mg/kg) or an equal volume of PBS on days 2 and 6. The mice were weighed daily. On the 9th day, all mice were euthanized, and their femurs and bloods were harvested for subsequent analysis.

### 2.5. Micro-Computed Tomography (Micro-CT) and Histopathology

The isolated femurs were fixed with 4% paraformaldehyde for one day. The distal metaphysis of the femur was analyzed by high-resolution micro-CT (NFR-Polaris-S160; Nanofocus Ray, Iksan, Korea) with a source voltage of 45 kVp, 90 µA current, and 7 µm isotropic resolution. Morphometric parameters, including bone volume per tissue volume (BV/TV), trabecular separation (Tb. Sp), trabecular thickness (Tb. Th), and trabecular number (Tb. N) were individually calculated using INFINITT-Xelis software 1.16 (INFINITT Healthcare, Seoul, Korea). For the bone histological analysis, femurs were dissected and fixed in 4% paraformaldehyde in PBS for 48 h. Femurs were then decalcified by placing them for two weeks in 12% EDTA, which was replaced every three days. The tissues were dehydrated, embedded in paraffin, and cut to a thickness of 6 μm along the coronal plate. The decalcified femoral sections were stained with hematoxylin and eosin (H&E) and tartrate-resistant acid phosphatase (TRAP).

### 2.6. Enzyme-Linked Immunosorbent Assay (ELISA)

Serum was collected from mice by cardiac puncture followed by centrifugation at 12,000× *g* for 15 min. The serum level of the bone resorption marker C-terminal telopeptide of type I collagen (CTX-I) was measured using ELISA kits (Mybiosource Inc., San Diego, CA, USA), according to the manufacturer’s instructions.

### 2.7. Preparation of Mouse Bone Marrow Macrophages (BMMs) and Osteoclast Differentiation

Bone marrow cells (BMCs) were isolated from 5-week-old ICR mice by flushing the tibias and femurs with growth medium α-MEM. To obtain BMMs, BMCs were seeded on culture dishes in α-MEM supplemented with 10% FBS and 10 ng/mL M-CSF and cultured for one day. Non-adherent cells were transferred to 10-cm petri dishes and further cultured in the presence of M-CSF (30 ng/mL) for three days. Cells attached to the bottom of the petri dishes were considered as BMMs. To differentiate BMMs into osteoclasts, BMMs were cultured in 48-well plates (3.5 × 10^4^ cells/well) in the presence of M-CSF (30 ng/mL) and RANKL (100 ng/mL) and the activity was tested by treatment with Vigeo (0, 25, 50, 100 μg/mL). After 3–4 days, the differentiated cells were fixed with 3.7% formalin for 15 min, permeabilized with 0.1% Triton X-100 for 10 min, and stained with a TRAP staining solution. The stained cells were observed under a microscope, and the number of TRAP-positive multinucleated cells (TRAP + MNCs) was counted.

### 2.8. Evaluation of Cytotoxicity

To determine cell viability by XTT assay, BMMs were seeded in 96-well plates (1 × 10^4^ cells/well) in the presence of M-CSF (30 ng/mL) with the indicated concentrations of Vigeo. Following 3-day incubation, XTT (sodium 30-[1-(phenyl-aminocarbonyl)-3, 4-tetrazolum]-bis(4-methoxy-6-nitro) benzenesulfonic acid hydrate and N-methyl dibenzopyrazine methyl sulfate) solution (50 µL) was added into each well and incubated for 4 h. Absorbance at 450 nm was measured using a multi-detection microplate reader (Molecular Devices, Sunnyvale, CA, USA).

### 2.9. Quantitative Real-Time Polymerase Chain Reaction (qRT-PCR)

Total RNA was extracted using TRIzol reagent (Thermo Fisher Scientific, Waltham, MA, USA) according to the manufacturer’s instructions and quantified by measuring the absorbance at 260 nm. Single-stranded cDNA was prepared from 1 μg of total RNA using M-MLV reverse transcriptase with oligo-dT primers according to the manufacturer’s instructions (Invitrogen, Carlsbad, CA, USA). Quantitative RT-PCR was carried out on the Exicycler 96 Real-Time Quantitative Thermal Block (Bioneer Co., Daejeon, Korea) using the SYBR^®®^ Green Premix (Bioneer Co., Daejeon, Korea) with specific primers. The amplification conditions were 95 °C for 5 min, followed by 40 cycles of 95 °C for 1 min, 60 °C for 30 s, and 72 °C for 1 min. Using *Gapdh* as an internal control, relative gene expression among samples was determined using the threshold cycle (C_t_) value. The primer sequences are listed in Table 1.

### 2.10. Filamentous-Actin (F-Actin) Assay

To experiment with F-actin ring formation, BMMs were incubated with M-CSF (30 ng/mL) and RANKL (100 ng/mL) at different concentrations of Vigeo (25, 50, and 100 μg/mL). After the 3-day incubation, the cells were fixed in 3.7% formalin for 15 min, permeabilized with 0.1% Triton X-100 for 10 min, with 0.25% bovine serum albumin (Sigma-Aldrich, St. Louis, MO, USA) for 30 min, and stained with phalloidin and a 40,6-diamidino-2-phenylindole (DAPI) solution (Life Technologies, Grand Island, NY, USA) to visualize F-actin and nuclei, respectively. Fluorescence was detected using a fluorescence microscope (Olympus FV1000, Tokyo, Japan) and images were analyzed using Image-Pro Plus software (version 4.0; Media Cybernetics, Silver Spring, MD, USA).

### 2.11. Resorption Pit Assay

The bone resorption assay was performed as described previously [21]. Briefly, mature osteoclasts were generated from the co-culture of BMCs and primary osteoblasts (OBs) in α-MEM complete medium containing 10^−8^ M Vitamin D3 and 10^−6^ M prostaglandin E2 (Sigma-Aldrich) for 10–12 days on collagen gel-coated culture dishes. Mature OCs were obtained by treatment with 0.1% collagenase type IV (Sigma-Aldrich, St. Louis, MO) and were reseeded in 48-well plates and dentin slices in the presence or absence of Vigeo (100 µg/mL). The cells re-seeded in dentin slices were completely removed using 10% sodium hypochlorite after 48 h. Subsequently, dentin slices were stained with hematoxylin. Bone slice images were taken using a microscope, and resorbed areas on bone slices were quantified with ImageJ software.

### 2.12. Western Blotting

Cells incubated with the indicated reagents for the indicated durations were washed with cold PBS and lysed with cold lysis buffer containing 50 mM Tris-HCl, 150 mM NaCl, 5 mM EDTA, 1% Triton X-100, 1 mM sodium fluoride, 1 mM sodium vanadate, 1% deoxycholate, and protease inhibitors on ice for 30 min. The lysate was collected after centrifugation at 10,000× *g* for 15 min. The protein concentration of each sample was quantified using a Bio-Rad DC Protein Assay Kit (Bio-Rad Laboratories Inc., Hercules, CA, USA). Equal amounts of proteins (10–20 µg) were separated by 10% SDS-PAGE, transferred to PVDF membranes (Bio-Rad, Hercules, CA), blocked with 5% nonfat milk in TBST buffer (100 mM NaCl, 10 mM Tris–HCl, pH 7.5, and 0.1% Tween-20) for 1 h at room temperature, and incubated with specific primary antibodies overnight at 4 °C. Finally, the membranes were incubated for 1 h at room temperature with the secondary antibodies. Following extensive washing in TBST, the specific protein bands were visualized using Immobilon Western Chemiluminescent HRP Substrate (Millipore, Billerica, MA, USA). Actin was used as a loading control.

### 2.13. Statistical Analysis

All quantitative data are presented as the mean ± standard deviation (SD) of at least three independent experiments. Statistical analysis was performed using Student’s *t*-test for comparisons between two groups and analysis of variance (ANOVA) followed by Tukey’s posthoc test for comparisons among three groups using GraphPad Prism 5 (GraphPad Software, San Diego, CA, USA). *p* < 0.05 was considered as statistically significant.

## 3. Results

### 3.1. Oral Administration of Vigeo Ameliorates LPS-Induced Bone Loss In Vivo

**Figure 1:**
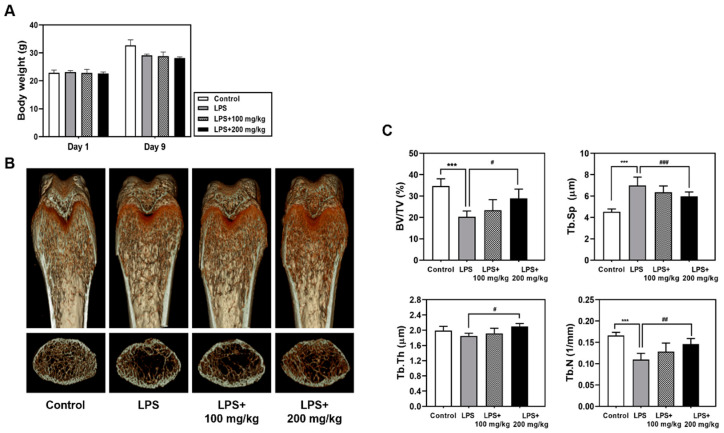
Vigeo attenuates lipopolysaccharide (LPS)-induced inflammatory bone loss. Mice were intraperitoneally injected with LPS (10 mg/kg) followed by treatment with indicated concentrations of Vigeo for 7 days. (**A**) Body weight measurement during the experimental period. (**B**) Representative three-dimensional micro-computed tomography (micro-CT) images of the coronal and transverse sections in the distal femurs of a mouse from each group. (**C**) Measurement of the trabecular morphometric parameters of bone volume/total volume (BV/TV), trabecular separation (Tb. Sp), trabecular thickness (Tb. Th), and trabecular number (Tb. N) in the proximal femurs using the micro-CT data using the INFINITT-Xelis software 1.16. Statistical analysis was performed using one-way analysis of variance (ANOVA) followed by Tukey’s posthoc test for comparisons among three groups. *** *p* < 0.01 versus the control group; ^#^ *p* < 0.05, ^##^ *p* < 0.01, ^###^ *p* < 0.001 versus the LPS group.

First, we investigated the effects of Vigeo on an LPS-induced bone loss mouse model. Mice were intraperitoneally injected with LPS and orally administered Vigeo or PBS. As shown in Figure 1A, all the experimental animals showed a stable increase in body weight during 9 days, demonstrating no biotoxicity at the Vigeo-treated concentration. As shown in Figure 1B, micro-CT analysis was performed using dissected femurs, which showed that LPS-induced mice developed a significant osteoporosis phenotype in femoral trabecular bone compared with those in the control group. In contrast, the group that was orally administered 200 mg/kg of Vigeo significantly reduced femoral trabecular bone loss in the LPS plus Vigeo group compared with that in the LPS alone group, which preserved the partial recovery of bone. Quantification of bone parameters verified that 200 mg/kg Vigeo administration significantly increased bone mineral density, such as BV/TV, Tb. N, and Tb. Th; whereas, Tb. Sp was decreased in LPS-induced bone loss mouse models (Figure 1C). In addition, the LPS plus Vigeo 100 mg/kg group also showed an inhibitory effect on bone loss but was not statistically significant. Taken together, the in vivo results indicated that Vigeo demonstrated considerable efficacy in rescuing bone loss.

### 3.2. Vigeo Effectively Attenuates LPS-Induced Osteoclast Activation In Vivo

**Figure 2:**
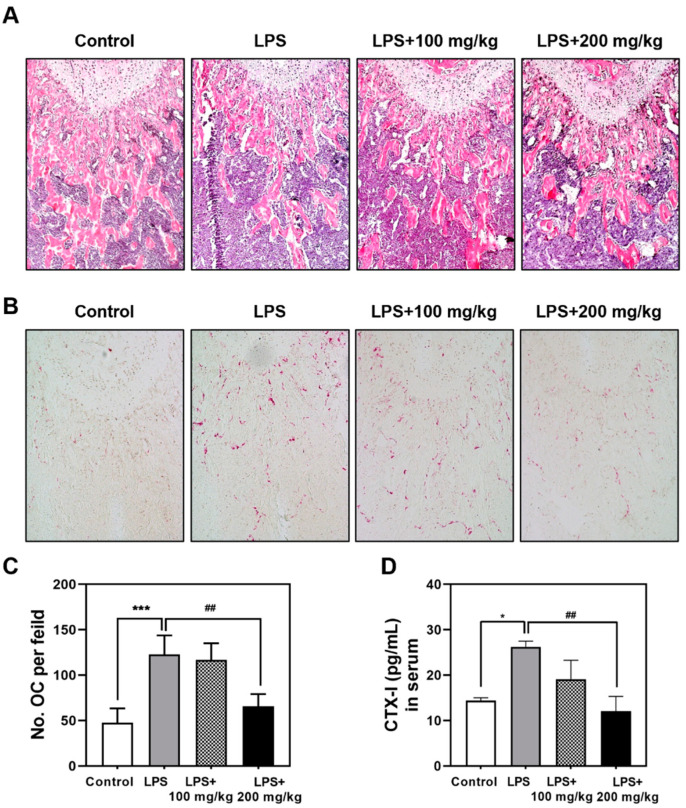
Vigeo inhibits lipopolysaccharide (LPS)-induced osteoclast activation in mice. (**A**) LPS injected mice were sacrificed after 9 days, and dissected femurs were fixed, decalcified, paraffin-embedded, and sectioned. The slices were stained with hematoxylin and eosin (H&E). (**B**) Slices stained with tartrate-resistant acid phosphatase (TRAP). (**C**) The number of osteoclasts per visual field of tissue was measured by histomorphometric analysis. (**D**) Blood samples were collected from mice and centrifuged to obtain serum for the evaluation of CTX-I levels by ELISA. Statistical analysis was performed using one-way analysis of variance (ANOVA) followed by Tukey’s posthoc test for comparisons among the three groups. * *p* < 0.05, *** *p* < 0.001 versus the control group; ^##^ *p* < 0.01 versus the LPS group.

Next, we confirmed the protective effect of Vigeo on histological changes of LPS-induced bone through H&E and TRAP staining. H&E staining showed severe bone loss and reduced bone volume with abnormal trabecular structures in the LPS-treated group (Figure 2A). However, treatment with Vigeo restored trabecular bone volume in the 200 mg/kg Vigeo-treated groups, showing restored trabecular density and preservation of bone structure (Figure 2A). Furthermore, TRAP staining results demonstrated that the LPS-treated group remarkably increased the number of TRAP-positive osteoclasts, while Vigeo attenuated LPS-induced osteolytic lesions, with decreased numbers of TRAP-stained mature osteoclasts (Figure 2B,C). As shown in Figure 2D, significant increase of CTX-I levels was observed in the LPS-induced group compared with the control group. Nonetheless, treatment with Vigeo reduced this tendency, as evidenced by the gradual decrease in CTX-I levels following Vigeo administration. These results reflect the effective anti-osteoclast activation effects of Vigeo in vivo.

### 3.3. Vigeo Inhibits Osteoclast Differentiation and Bone Resorption In Vitro

**Figure 3:**
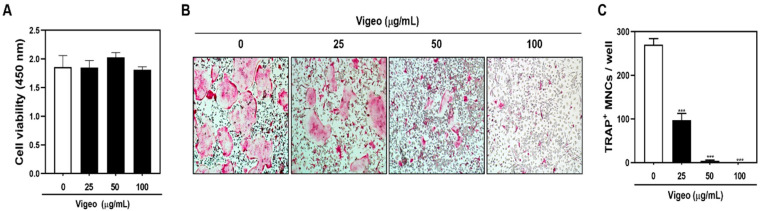
Vigeo attenuates receptor activator of nuclear factor-B ligand (RANKL)-induced osteoclast differentiation without cytotoxicity. (**A**) Bone marrow macrophages (BMMs) were cultured with 30 ng/mL M-CSF and the indicated concentration of Vigeo for 3 days. Cell viability of Sec was evaluated using the XTT assay by measuring absorbance at 450 nm. (**B**) BMMs were seeded with M-CSF (30 ng/mL) and stimulated with RANKL (100 ng/mL) and the indicated concentrations of Vigeo for 3 days. TRAP staining was performed to assess osteoclast formation and representative images were captured. (**C**) TRAP-positive multinucleated cells counted as osteoclasts (nuclei > 5). *** *p* < 0.001 versus the control group.

**Figure 4:**
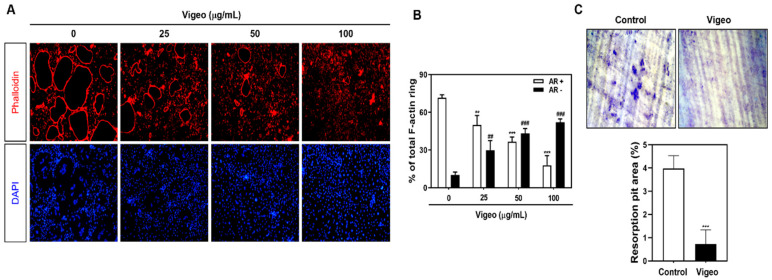
Vigeo suppresses the F-actin ring formation and bone-resorbing activity of mature osteoclasts. (**A**) Bone marrow macrophages (BMMs)were cultured with M-CSF (30 ng/mL) and receptor activator of nuclear factor-B ligand (RANKL) (100 ng/mL) in the presence of the indicated concentrations of Vigeo. The cells were fixed, permeabilized, and stained with phalloidin and 4′,6-diamidino-2-phenylindole (DAPI). The cells were examined under a confocal laser scanning microscope. (**B**) Normal actin ring (AR+) osteoclasts and disrupted actin ring (AR−) osteoclasts were counted and compared with the total number of osteoclasts that contain three or more nuclei. The graph shows the comparison between relative percentage of osteoclasts that express AR+ and AR−. ** *p* < 0.01 and *** *p* < 0.001 versus AR+ osteoclasts in control group; ^##^ *p* < 0.01 and ^###^ *p* < 0.001 versus AR− osteoclasts in control group. (**C**) Mature osteoclasts from the co-culture system were seeded in dentin slices for 48 h with or without Vigeo (100 μg/mL). Cells attached to the hydroxyapatite-coated plate were removed, and the plates were photographed under a light microscope. *** p < 0.001 versus the control.

In order to prevent Vigeo-induced toxicity in primary BMM cells, we determined the nontoxic concentrations of Vigeo against preosteoclasts to observe its anti-osteoclastogenesis effects. The XTT assay results showed that Vigeo had no significant effect on cell viability at the indicated concentrations (Figure 3A). Next, we assessed the anti-osteoclastogenetic effect of Vigeo by culturing BMM cells with RANKL and M-CSF in the presence and absence of Vigeo and then examined them after TRAP staining. The results showed the formation of osteoclasts in control groups after RANKL stimulation, whereas the addition of Vigeo remarkably delayed the differentiation of osteoclasts in a dose-dependent manner (Figure 3B,C), indicating that nontoxic levels of Vigeo were able to significantly suppress osteoclast formation. Osteoclast function requires a well-formed F-actin rings, which is consistent with the results shown in Figure 4A,B. Upon stimulation with RANKL, BMMs showed the generation of the characteristically polarized F-actin ring. However, treatment with Vigeo markedly diminished the number and size of F-actin rings, demonstrating inhibited osteoclast activation (Figure 4A,B). To further test the effects of Vigeo on RANKL-induced osteoclast bone resorbing function, we performed a pit formation assay on dentin slices. As shown in Figure 4C, Vigeo treatment effectively decreased the number of resorption pits. These results collectively demonstrate that Vigeo reduced the formation and bone resorption properties of osteoclasts without cytotoxic effects.

### 3.4. Vigeo Inhibits RANKL-Activated Osteoclast Differentiation Signaling Pathways and Osteoclast Specific Genes In Vitro

**Figure 5:**
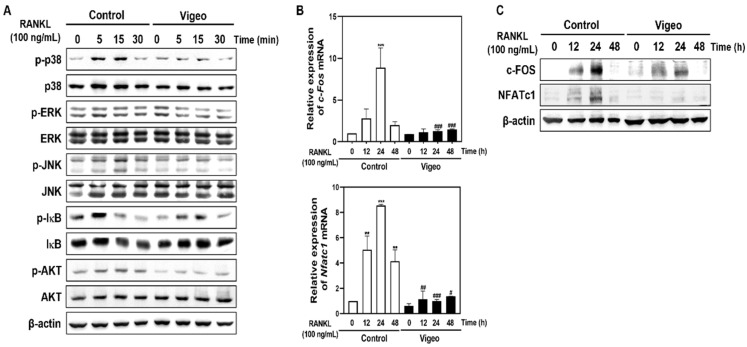
Vigeo affects receptor activator of nuclear factor-B ligand (RANKL)-induced phosphorylation of early signals and expression of c-FOS and NFATc1. (**A**) Bone marrow macrophages (BMMs)were seeded in the presence of M-CSF (10 ng/mL) for 1 day and then starved for 4 h. (**A**) Vigeo was used to pretreat the cells for 1 h followed by 0, 5, 15, and 30 min of RANKL stimulation. Whole-cell proteins were extracted by lysis using RIPA buffer and the protein expression was detected by western blotting using the indicated antibodies. (**B**) BMMs were cultured with M-CSF (30 ng/mL) and RANKL (100 ng/mL) in the presence or absence of Vigeo (100 μg/mL). Total RNA was isolated from the cells and mRNA expression levels of *c-Fos* and *Nfatc1* were evaluated by real-time RT-PCR. ** *p* < 0.01, *** *p* < 0.001 versus the control; ^#^ *p* < 0.05, ^##^ *p* < 0.01, ^###^ *p* < 0.001 versus the control at indicated times. (**C**) BMMs were cultured with M-CSF (30 ng/mL) and RANKL (100 ng/mL) in the presence or absence of Vigeo (100 μg/mL). Protein expression was detected using western blotting with the indicated antibodies. β-actin was used as an internal control.

**Figure 6:**
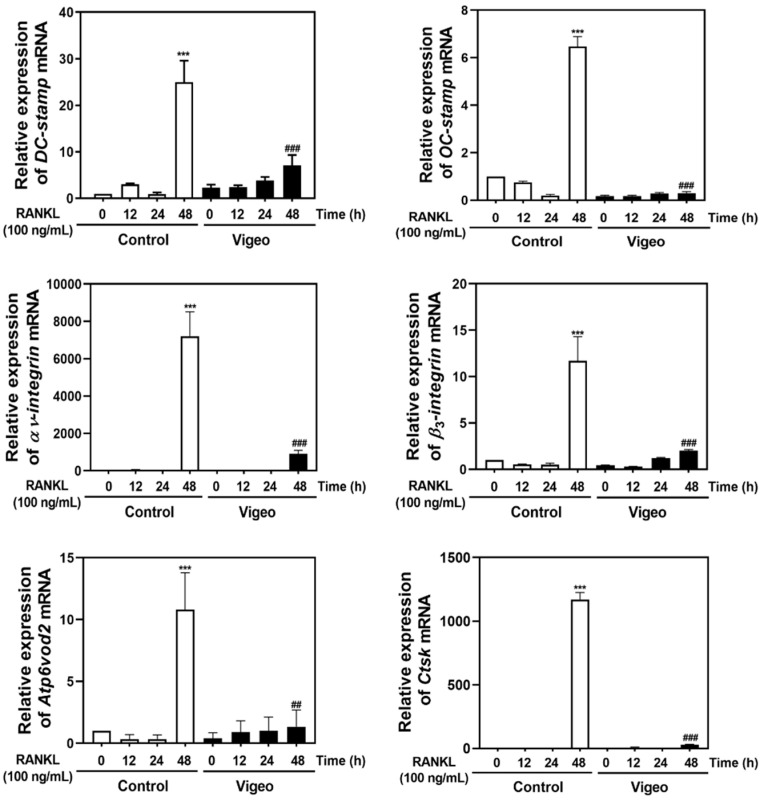
Vigeo inhibits receptor activator of nuclear factor-B ligand (RANKL)-induced mRNA expression of *DC-stamp*, *OC-stamp*, *αv-integrin*, *β_3_-integrin*, *Atp6v0d2*, and *CtsK*. Bone marrow macrophages (BMMs)were incubated with M-CSF (30 ng/mL) and RANKL (100 ng/mL) in the presence or absence of Vigeo (100 μg/mL) for the indicated time. Total RNA was isolated from cells using TRIzol reagent, and the mRNA expression of *DC-stamp*, *OC-stamp*, *αv-integrin*, *β_3_-integrin*, *Atp6v0d2*, and *CtsK* was analyzed by real-time RT-PCR. *** *p* < 0.001 versus the control at 0 h; ^##^ *p* < 0.01, ^###^ *p* < 0.001 versus the control at 48 h.

To elucidate the upstream mechanisms by which Vigeo affects osteoclast activation, we investigated the AKT, IκB, and MAPK (JNK, p38, and ERK) signaling pathways, and their downstream molecules NFATc-1 and c-FOS. As shown in Figure 5A, increased phosphorylation of AKT, JNK, p38, ERK, and IκB mediated by RANKL stimulation was significantly blocked by Vigeo. Next, to evaluate whether Vigeo affect the induction of *c-Fos*, *Nfatc1*, and osteoclast-specific genes, we examined their mRNA and protein levels. As shown in Figure 5B, Vigeo suppressed RANKL-induced expression of *c-Fos* and *NfatC1* mRNA. In agreement with these results, the protein levels of *c-Fos* and *Nfatc1* induced by RANKL were significantly increased, and this increase was significantly inhibited by Vigeo (Figure 5C). In addition, Vigeo also inhibited the RANKL-induced increase in the mRNA expression of *DC-cstamp*, *OC-stamp*, *αv-integrin*, *β_3_-integrin*, *Atp6v0d2*, and *Ctsk* at 48 h (Figure 6). These results demonstrate that Vigeo inhibited RANKL-induced osteoclastogenesis through de-phosphorylation of AKT, JNK, p38, ERK, and IκB, followed by downregulation of transcription factors.

## 4. Discussion

Fermentation is a biological system that depended on the microbial conversion of complex structures into simple compound by microorganisms such as bacteria, yeast, and fungi [8]. Fermentation-mediated bioactivation of plant medicinal products results in improved therapeutic efficacy and decreased toxicity [8,22], and fermentation is also known to have beneficial effects on the absorption and bioavailability of plant products by facilitating the production of active components into their metabolites or by generating low-molecular-weight substances such as aglycones from glycosides [23,24]. These substances have diverse biological activities, such as anti-inflammatory activity, anti-infection, and anti-cancer properties [25,26]. Nuruk is a traditional Korean fermentation starter used to produce alcoholic beverages using various grains [27]. Nuruk contains different types of microorganisms, which are responsible for saccharification and alcoholic fermentation [27]. Recent studies have demonstrated that treatment with Nuruk extract is associated with a decrease in LPS-induced nitrite and IL-6 levels in RAW 264.7 cells [28], and also has an inhibitory effect on hypertension, migration, platelet aggregation, and angiogenesis [29].

In this study, we demonstrated for the first time that Vigeo, a functional extract using the traditional nuruk fermentation method, can inhibit RANKL-induced osteoclastogenesis by blocking the AKT, NF-κB and MAPK signaling pathways in vitro and prevent bone loss in vivo in an LPS-triggered bone loss mouse model. LPS-treated mice showed inflammatory bone loss by stimulating osteoclast differentiation, while oral-administration of Vigeo in LPS-treated mice prevented these effects. The micro-CT analysis results showed that LPS-treated mice had suppressed BV/TV ratio, increased Tb. Sp (μm), and suppressed Tb. N (per mm) and Tb. Th (μm); the effects that were significantly reversed by Vigeo treatment. In addition, the histological features were improved by Vigeo treatment in LPS-treated mice, and the LPS-treated TRAP-positive osteoclast count was also suppressed by Vigeo. Furthermore, Vigeo suppressed the levels of the bone resorption marker CTX-1 in the serum of LPS-treated mice.

Monocytes and macrophages derived from hematopoietic stem cells of the bone marrow are differentiated during the maturation step of osteoclastogenesis (differentiation, proliferation, polarization, and resorption) to generate osteoclasts. This process is regulated by the major cytokines M-CSF and RANKL, which differentiate BMM osteoclast precursors into osteoclasts [30]. Through TRAP staining and measuring the levels of bone resorption activity, such as the formation of F-actin and resorption pits, we determined that osteoclastogenesis and differentiation function can be effectively induced by co-stimulation with M-CSF and RANKL. The present study shows that non-toxic concentrations of Vigeo could inhibit large number of RANKL-stimulated TRAP-positive osteoclasts, demonstrating that Vigeo showed effective inhibitory effects against RANKL-induced osteoclastogenesis. Furthermore, the bone resorption activity, as measured by the materialization of F-actin ring structures, was also suppressed by Vigeo via inhibition of the formation of resorption pits formed by RANKL stimulation. These findings were further demonstrated by the reduced formation of F-actin rings and bone resorption pits which allow us to further explore the underlying mechanisms and to identify new molecular targets for the treatment of osteoporosis in the future.

Previous studies have revealed the biological effects of ESM extracts, a major component of Vigeo, including anti-inflammatory [10], anti-tumor [31], anti-steatosis [32], and neuroprotective [33] effects. Specifically, ESM extracts were found to inhibit MAPK and NF-κB signaling pathways, which are closely associated with osteoclast formation [12]. In the present study, Vigeo inhibited the phosphorylation of p38, ERK, JNK, IκB, and AKT in osteoclast precursor cells. Altogether, these results indicate that Vigeo could suppress the activities of *c-Fos* and *Nfatc1* along with other transcriptional regulators of osteoclastogenesis. In addition, the regulation of *c-Fos* and *Nfatc1* by Vigeo during osteoclastogenesis is promoted by the activity of osteoclast-specific genes such as *DC-stamp*, *OC-stamp*, *αv-integrin*, *β_3_-integrin*, *Atp6v0d2*, and *Ctsk*. In future research, we will carry out a comparative analysis of the activity of a mixture of ESM, AJN, and AJK, which are constituents of Vigeo, and traditional nuruk fermented extract. Moreover, we will conduct isolation, purification, and analysis of components of Vigeo, which consists of various physiologically active substances.

## 5. Conclusions

Our findings show that Vigeo protects against LPS-induced inflammatory bone loss by inhibiting osteoclast activity in vivo. In vitro mechanistic studies demonstrated that Vigeo inhibits not only osteoclast differentiation but also F-actin formation and bone resorption through the inhibition of MAPK, NF-κB, and AKT signaling, which leads to *c-Fos* and *Nfatc1* activation and expression of downstream genes. Although additional preclinical studies and clinical trials are needed to evaluate safety and efficacy in human, this study is the first to support the use of Vigeo as a promising treatment for the prevention of inflammatory bone diseases.

## Figures and Tables

**Table 1:** Primer sequences used for real-time qRT-PCR analysis.

Gene Name	Sequence (5′-3′)	
	Forward	Reverse
*Gapdh*	TCAAGAAGGTGGTGAAGCAG	AGTGGGAGTTGCTGTTGAAGT
*c-Fos*	GGTGAAGACCGTGTCAGGAG	TATTCCGTTCCCTTCGGATT
*Nfatc1*	GAGTACACCTTCCAGCACCTT	TATGATGTCGGGGAA AGAGA
*DC-stamp*	TCCTCCATGAACAAACAGTTCCA	AGACGTGGTTTAGGAATGCAGCTC
*O* *C-* *stamp*	ATGAGGACCATCAGGGCAGCCACG	GGAGAAGCTGGGTCAGTAGTTCGT
*αv-integrin*	ACAAGCTCACTCCCATCACC	ATATGAGCCTGCCGACTGAC
*β_3_-integrin*	GGAGTGGCTGATCCAGATGT	TCTGACCATCTTCCCTGTCC
*Atp6v0d2*	GACCCTGTGGCACTTTTTGT	GTGTTTGAGCTTGGGGAGAA
*Cathepsin K (Ctsk)*	CCAGTGGGAGCTATGGAAGA	CTCCAGGTTATGGGCAGAGA

## Data Availability

The data used to support the finding of this study are available from the corresponding author upon request.
